# Dendritic cell vaccines containing lymphocytes produce improved immunogenicity in patients with cancer

**DOI:** 10.1186/s12967-014-0338-3

**Published:** 2014-12-05

**Authors:** Mayu O Frank, Julia Kaufman, Salina Parveen, Nathalie E Blachère, Dana E Orange, Robert B Darnell

**Affiliations:** Laboratory of Molecular Neuro-oncology, The Rockefeller University, 1230 York Avenue, New York, NY 10065 USA; Howard Hughes Medical Institute, Laboratory of Molecular Neuro-Oncology, The Rockefeller University, New York, NY 10065 USA; New York Genome Center, 101 Avenue of the Americas, New York, NY 10013 USA; Hospital for Special Surgery, 535 East 70th Street, New York, NY 10021 USA

**Keywords:** Dendritic cells, Vaccine, Lymphocytes, Immunogenicity, Cancer, Adjuvant

## Abstract

**Background:**

Dendritic cells are currently under investigation for their ability to generate anti-cancer immune responses. No consensus has been reached as to the optimal method of dendritic cell vaccine preparation and is a barrier to success in the field.

**Methods:**

Over a course of three separate dendritic cell vaccine studies to treat cancer, we tested two different methods for preparing dendritic cells from peripheral blood mononuclear cells: adherence and antibody-selected CD14+ cells.

**Results:**

Surprisingly, we found that patients who received dendritic cell vaccines generated by the adherence method mounted increased T cell proliferation in response to vaccination. This difference could not be accounted for by dendritic cell vaccine dose, cell surface phenotype or dendritic cell function *in vitro*. One notable difference between the two vaccine preparation methods was that the dendritic cell vaccine cultures generated by the adherence method contained up to 10% lymphocytes, and these lymphocytes were proliferating and producing IFNγ in response to antigen *in vitro* at the time of administration.

**Conclusions:**

Enhanced immunogenicity of adherence dendritic cell vaccinations may be due to the presence of lymphocytes during dendritic cell culture.

**Trial registration:**

Clinicaltrials.gov identifiers: NCT00289341, NCT00345293, and NCT00893945

**Electronic supplementary material:**

The online version of this article (doi:10.1186/s12967-014-0338-3) contains supplementary material, which is available to authorized users.

## Background

Dendritic cells (DCs) are potent antigen presenting cells (APCs), which prime and activate CD4 helper [1] and CD8 cytotoxic killer T cells [2] and are therefore useful for initiating cancer specific immune responses. In fact, at the time of this writing, there are 417 studies listed on clinicaltrials.gov retrieved with the search terms, “dendritic cell” and “cancer”. Despite 20 years of DC vaccine trials, clinically meaningful responses have been sparse. In the search for more potent DC vaccines, different methods of precursor isolation, cell differentiation, antigen pulsing, maturation and the use of adjuvants are being evaluated. While the use of peripherally circulating monocytes as precursors and IL-4 and GM-CSF for differentiation have become an accepted standard [3,4], other areas of DC vaccine production remain highly variable [3,5].

In our own studies, we have explored the use of apoptotic tumor cells as a source of antigen for DCs. This concept arose from the finding that patients with paraneoplastic neurologic disorders (PND) can exhibit potent, naturally occurring tumor immunity and harbor antigen specific T cells to neuronal antigens [6]. At the same time, while DCs are required to initiate de novo T cell responses, the source of antigen for the DCs was unknown, as DCs do not express the neuronal PND antigens. These observations, together with those of Rosen and colleagues regarding the effects of UV irradiation on the packaging of Lupus auto antigens into membrane-bound bodies [7], led us to the hypothesis that PND antigens were transferred from apoptotic tumor cells to the phagocytic DCs, and then presented to naïve T cells to trigger a potent immune response [8]. This hypothesis was borne out with a model antigen (influenza) and with PND antigen (cdr2) [6] establishing a basis for the phenomenon of cross-priming proposed by Bevan [9,10].

Here we tested the immunogenicity of DCs presenting apoptotic tumor cells in patients with cancer, using 2 different methods of isolating DC precursors: adherence of peripheral blood mononuclear cells (PBMCs) to plastic (Adherence DCs) and selection of CD14+ cells using antibody-conjugated beads by CliniMACS (Selected DCs). In all studies, vaccines were prepared by culturing DC precursor cells with IL-4 and GM-CSF for 6 days, after which immature DCs (iDCs) were cultured with apoptotic tumor cells and treated with maturation stimulus cocktail of TNF-α, prostaglandin E2, plus or minus CD40L. Our first clinical trial of a DC vaccine treatment for prostate cancer included 24 patients who were treated with Adherence DCs pulsed with apoptotic LNCaP, a prostate cancer cell line [11]. Proliferation responses were assessed by ^3^H thymidine incorporation assay pre- and post-vaccination and responses to the prostate cell lines LNCaP and PC3 were determined. Treatment induced a statistically significant increase in T cell proliferative responses to both prostate tumor cell *in vitro*.

In a second follow up trial, in an effort to make a more potent vaccine, two modifications were made to the vaccine. First, DCs were made using the selection method, assuming that there may be benefits to using a purer vaccine and second, a different prostate cancer cell line, PC3, was used to pulse the DCs. In our first study in which patients were vaccinated with DC/LNCaP, post-vaccination proliferation responses to PC3 (to which patients were not vaccinated) were equivalent to LNCaP. Though this was unexpected because the PC3 cells do not express known prostate cancer antigens such as PSA and PSMA, this result led us to hypothesize that PC3 is a more immunogenic cell line. Therefore, in a follow up study, seven patients were enrolled and vaccinated with Selected DCs pulsed with apoptotic PC3. Surprisingly, as we shall describe, the lymphocyte proliferation responses post-vaccination were markedly lower than that had been previously observed in the first study. This difference could not be explained by the amount of vaccine administered or the maturation status of the DCs. However, it was not clear whether this observation was due to the change in prostate cancer cell line used (LNCaP versus PC3) or the change in the method of DC preparation (Adherence versus Selected). To test this, we enrolled 3 subsequent patients who were given Adherence DC pulsed with PC3 to assess whether the change in the method of DC preparation was the cause of the difference in proliferation. Here, we compare *in vitro* phenotypic and functional similarities and differences between the two vaccine preparation methods.

## Methods

### Patients and study design

Two separate DC vaccination studies were conducted for prostate cancer patients, the first using apoptotic LNCaP cells, as reported previously [11], and the second using apoptotic PC3 cells. Patients in the first study were vaccinated with DCs pulsed with LNCaP (DC/LNCaP) and DCs pulsed with LNCaP transfected with influenza M1 protein (DC/LNCaP-M1) [11]. In the second study patients were vaccinated with DCs pulsed with PC3 (DC/PC3) and DCs pulsed with PC3 transfected with influenza M1 (DC/PC3-M1). The ratio of DCs to apoptotic LNCaP or PC3 tumor cells was 1:1. In both studies, the acceptable dose range was 1-10^6^ DCs of each type at each time point, regardless of the method of DC preparation. Patients in both studies were also given DCs pulsed with KLH (DC/KLH) as a control antigen. Initial vaccination was followed by 3 booster vaccine immunizations, each 2 weeks apart, administered subcutaneously. In both studies, leukocytes for immunomonitoring were collected by leukapheresis at baseline and again 6 weeks after the last booster.

In a third study, patients with primary brain tumors were vaccinated with DCs pulsed with autologous apoptotic tumor cells and DC/KLH. In this study, both the number of boosters and the timing of the post-vaccination leukapheresis were different from the first 2 studies. Patients were vaccinated with either 2 or 3 doses every 3 weeks intradermally and leukapheresed 2 to 3 weeks after the 2^nd^ dose. Here, we report only the patients’ responses to DC/KLH as relevant and not the responses to the DC vaccine to brain tumor. In all 3 studies, the first dose of DC administered was “fresh” and all subsequent booster doses were thawed doses. All studies were conducted at Rockefeller University Hospital after Institutional Review Board approval. Written consent was obtained from all patients. Study identifiers on clinicaltrials.gov were: NCT00289341, NCT00345293, and NCT00893945.

### Adherence method of dendritic cell vaccine preparation (Adherence DCs)

DCs were prepared as previously described [11]. Briefly, leukapheresates were placed over lymphocyte separation media and the buffy layer was collected and washed. PBMCs were then plated in RPMI-1640 supplemented with 1% autologous plasma and allowed to adhere at 37°C. After 1 hour, the non-adherent cells were removed. The adherent cells were differentiated in RPMI-1640 supplemented with 1% autologous plasma, GM-CSF (Genzyme) and IL-4 (R & D Systems) over 6 days, at which point they are non-adherent and considered immature DCs. LNCaP and PC3 cells were obtained directly from American Type Cell Culture (CRL-1740 and CRL-1435) and cell banks were established as described [11]. LNCaP or PC3 cells were UV irradiated and cultured with immature DCs at a 1:1 ratio with PGE_2_ (Sigma) and TNFα (R & D Systems, Miltenyi) over 36-48 hours. A subset of immature DCs was cultured with KLH (biosyn). The cells were harvested on the 8^th^ day, washed and resuspended in 5% dimethyl sulfoxide and 10% human serum albumin (HSA, Grifols) in normal saline for freezing or administration.

### Selection method of dendritic cell vaccine preparation (Selected DCs)

Leukapheresates were washed with PBS/EDTA supplemented with 2% HSA, incubated with CD14 MicroBeads (Miltenyi Biotec) for 15 minutes then washed. CD14+ cells were isolated using the CliniMACS System (Miltenyi Biotec). Positively selected cells were washed and plated in RPMI-1640 supplemented with 1% autologous plasma, GM-CSF (Genzyme) and IL-4 (R & D Systems) over 6 days. Cells were pulsed with tumor cells, matured and harvested in the same manner as Adherence DCs.

### Lymphocyte proliferation assay

Lymphocyte proliferation responses, pre- and post-vaccination, were measured by ^3^H-thymidine incorporation assays as previously described [12] with the following changes. APCs were either pre-vaccination CD14+ cells or DCs. ^3^H-thymidine was added for the last 20 hours of culture. Results are presented as average count per minute (CPM) of 3 to 6 replicate wells.

### Phenotyping by flow cytometry

As part of release criteria testing, DCs were counted and stained with HLA-DR, CD14, CD83 antibody (Becton Dickinson) and propidium iodide (Serologicals). For additional phenotyping, cells were stained with CD86, CD40, CCR7, CD4, CD8, CD69, and CD19 antibody (Becton Dickinson). Data was acquired on the FACSCalibur (Becton Dickinson) or MACSQuant VYB (Miltenyi Biotec) and data were analyzed using FlowJo software.

### Phagocytosis assay

Immature Selected or Adherence DCs were stained with HLA-DR-APC and cultured with apoptotic PKH26 stained allogeneic lymphocytes with or without EDTA for 24 hours. Analysis was restricted to HLA-DR+ cells and the phagocytosis of apoptotic cells by DC groups were identified by positive PKH26 dye staining.

### Allogeneic mixed leukocyte reaction (Allo-MLR)

As previously described [12], DCs were cultured with the non-adherent fraction of allogeneic PBMCs.

### Cytokine analysis

Supernatants of Adherence and Selected DCs were assessed for the presence of cytokines using the Meso Scale Discovery Human ProInflammatory 9-Plex plate and read on the SECTOR Imager (Meso Scale Discovery). DCs were prepared using each of the 2 methods from 3 donors.

### IFNγ ELISPOT

The IFNγ ELISPOT assay was done as previously described [12] with the following modifications. Influenza-infected DCs (DCF) were used to elicit influenza-specific IFNγ responses. Adherence DC vaccine preparations were harvested on day 8 and re-plated on an IFNγ ELISPOT coated plate at a density of 25,000 lymphocytes/well.

### Dextramer staining and intracellular flow cytometry analysis

PBMCs or Adherence DC preparations from A0201 donors were co-cultured with apoptotic 3T3 (DC/ctrl) or apoptotic influenza-infected 3T3 cells (DC/flu) for 5 hours in GolgiStop (BD Bioscience) before staining with APC-conjugated A0201 Influenza-M1 dextramer (GILGFVFTL, Immudex) followed by CD8-FITC (BD Bioscience). Cells were fixed and permeabilized (Cytofix/Cytoperm, BD Bioscience) as per manufacturer’s protocol. Intracellular cytokine staining was performed using IFNγ − PE (BD Bioscience) followed by flow cytometry analysis.

### Statistical analysis

To evaluate the difference between proliferation responses between the patients given Selected DCs and Adherence DCs, the Mann-Whitney test was used. This test was also used to describe differences in the number and phenotype of the 2 types of DCs given. The paired t-test was used to describe differences in all other assays. P-values less than 0.05 were considered statistically significant.

## Results

### Adherence DCs elicit increased proliferation of lymphocytes post-vaccination

**Figure 1:**
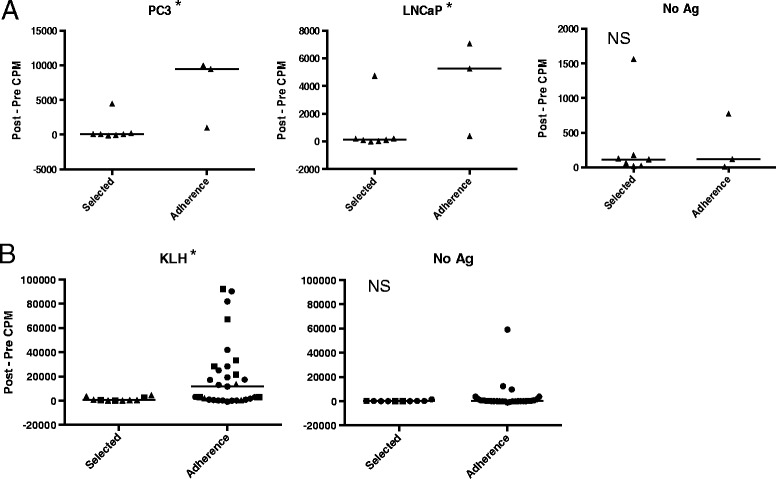
**Adherence DCs elicit increased proliferation of lymphocytes post-vaccination. (A)** ^3^H thymidine proliferation responses to prostate cancer cells lines, PC3, LNCaP or no antigen of lymphocytes from patients vaccinated with either Selected or Adherence DCs pulsed with PC3. CD14+ monocytes were used as APCs. Results are presented as average pre-vaccination counts per minute (CPM) subtracted from average post-vaccination CPM for each antigen group. The results are the average of 3 to 6 replicate wells. Black line indicates median. * indicates p < 0.05. NS = not statistically significant. **(B)** Pooled ^3^H thymidine proliferation responses to KLH or no antigen of lymphocytes from patients vaccinated with either Selected or Adherence DCs from 3 clinical trials. ● indicates response of patient in DC/LNCaP study, **▲** indicates response of patient in DC/PC3 study, and ■ indicates response of patient in DC pulsed with autologous brain tumor study.

Seven patients received Selected DCs and three received Adherence DC vaccine preparations, all pulsed with apoptotic PC3 cells. We compared the post- minus pre-vaccination proliferation responses of these patients. There was a significantly higher proliferation response to PC3 in patients that received Adherence DCs as compared to those who received Selected DCs (Figure 1A, p = 0.033; Additional file 1: Figure S1). Similarly, there was a significantly higher proliferation response in the Adherence versus the Selected DC vaccines to LNCaP, another prostate cancer cell line with which this cohort was not vaccinated (p = 0.033). Background post-vaccination proliferation responses to APCs not pulsed with antigen did not differ between the patients receiving Adherence or Selected DC/PC3 vaccines (No Ag, p = 0.867). This data indicated that the DC/PC3 vaccines prepared by Adherence method elicited higher antigen specific immune responses than those prepared by the Selection method.

This observed difference in immunogenicity was not anticipated. Therefore, we confirmed this by pooling data from other DC vaccine trials conducted by our laboratory, including the previously reported study in prostate cancer patients using DC/LNCaP and an additional DC vaccine study of patients with primary brain tumors. Since the tumor type was different between these studies, tumor specific responses could not be reasonably compared. However, all patients were vaccinated with DC/KLH made with either Selected or Adherence DCs, therefore, proliferation responses to KLH were examined. There were a total of 10 evaluable patients who received Selected DC/KLH and 31 patients who received Adherence DC/KLH. The vaccination induced significantly higher lymphocyte proliferation responses to KLH in patients that received Adherence DCs than those that received Selected DCs (Figure 1B; p = 0.023). No difference was observed in the proliferative lymphocyte responses to negative control APCs (not pulsed with antigen, p = 0.576). This result indicates that Adherence DCs consistently elicit higher immunogenicity even in different populations of cancer patients.

The differences in immunogenicity were not a result of differences in DC dose, phenotype, such as CD83 or CD14 expression or a result of differences in DC viability. The total number of DC/PC3, DC/PC3-M1, and DC/KLH administered also did not differ between Adherence and Selected vaccine preparations (Table 1). Within the second study of DC loaded with apoptotic PC3, the number of cells administered, the cell surface expression of CD83 and CD14, and viability of DC/PC3 and DC/PC3-M1 were similar. Further, in all three studies (DC/LNCAP, DC/PC3 and DC/autologous brain tumor), these same parameters are also similar in the DC/KLH that were administered. Given such similarities between Adherence and Selected DCs, DC phenotype, viability, and dose do not explain the difference in immunogenicity.

**Table 1:** **Number and phenotype of DCs administered**

**Median number of DCs administered per patient**							
		no. x 10^6^	(range)				
DC/PC3	Selected	28.15	(23.17-37.10)				
	Adherence	30.73	(25.90-31.84)				
	p-value	>0.99					
DC/PC3-M1	Selected	28.86	(20.49-33.85)				
	Adherence	26.75	(24.80-34.05)				
	p-value	0.80					
**Phenotype of DCs administered (median)**							
		CD14+ (%)	(range)	CD83+ (%)	(range)	PI+ (%)	(range)
DC/PC3	Selected	2.76	(1.85-5.71)	96.18	(86.35-98.46)	7.94	(4.36-18.06)
	Adherence	1.09	(0.82-4.41)	97.70	(90.70-98.40)	8.99	(4.81-11.50)
	p-value	0.23		>.99		0.87	
DC/PC3-M1	Selected	3.45	(1.15-7.9)	95.60	(85.67-98.49)	12.52	(4.34-17.09)
	Adherence	2.04	(1.29-6.4)	98.20	(96.80-98.90)	10.10	(5.83-12.60)
	p-value	0.87		0.12		0.72	
**Median number of DC/KLH administered per patient in all three studies**							
		no. x 10^6^	(range)				
	Selected	9.04	(5.06-17.41)				
	Adherence	9.86	(4.69-16.31)				
	p-value	0.59					
**Phenotype of DC/KLH administered in all three studies (median)**							
		CD14+ (%)	(range)	CD83+ (%)	(range)	PI+ (%)	(range)
	Selected	0.64	(0.01-2.00)	96.79	(92.35-99.30)	1.85	(0.92-9.30)
	Adherence	0.36	(0-2.82)	95.69	(86.00-99.97)	1.27	(0.34-12.19)
	p-value	0.24		0.55		0.10	

### In vitro assays of DC phenotype and function do not predict differences in immunogenicity

**Figure 2:**
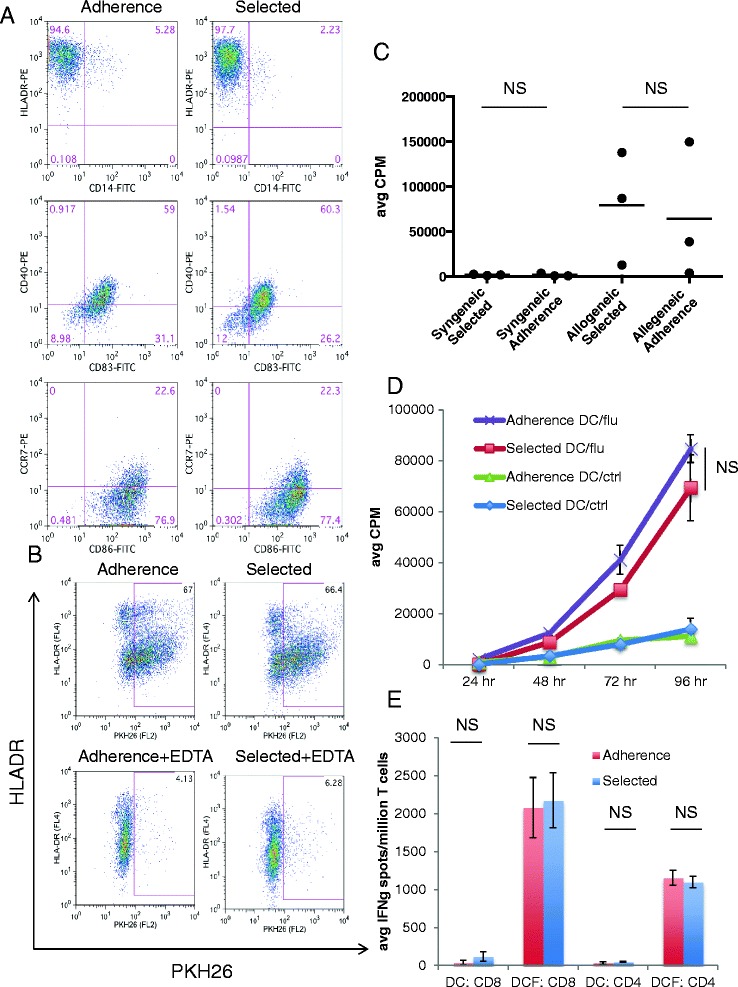
**In vitro assays of phenotype and function do not predict differences in immunogenicity. (A)** Cell surface marker staining. Selected or Adherence DCs made from the same donor were stained with CD14, CD83, HLA-DR, CD86, CD40 and CCR7 antibody. This is representative of 3 repeated experiments. **(B)** Phagocytosis assay. HLA-DR stained Selected or Adherence iDCs were cultured with PKH26 stained apoptotic lymphocytes with or without EDTA for 24 hours. Cells are gated on HLA-DR. Cells staining positive for both HLA-DR and PKH26 indicate phagocytosis of apoptotic cells by DC groups. The data shown is representative of 3 repeated experiments. **(C)** Allo-MLR. Selected and Adherence DCs were made from cells from 3 donors. Average CPMs are shown for syngeneic and allogeneic responses at the DC:T cell ratio of 1:30. Solid black line represents the mean. NS = not statistically significant. **(D)** Lymphocyte proliferation. Adherence or Selected DCs co-cultured with apoptotic 3T3 cells (DC/ctrl) or with influenza-infected 3T3 cells (DC/flu), were cultured with syngeneic CD14- cells. The cultures were assessed for proliferation by ^3^H thymidine incorporation and the data shown is representative of 3 repeated experiments. NS = not statistically significant. **(E)** IFNγ ELISPOT. Purified syngeneic CD8 or CD4 T cells were plated in an ELISPOT with either non-infected DCs (DC) or influenza-infected (DCF) made by the 2 methods. The data shown is an average of triplicate wells and representative of 3 repeated experiments. NS = not statistically significant.

To test whether Adherence or Selected DCs express different levels of markers of maturation, DCs were prepared using both methods from the same donor’s PBMC. Adherence and Selected DCs were assessed for the surface markers CD14, HLA-DR, CD83, CD86, CD40 and CCR7, but no differences were found (Figure 2A). We next evaluated the *in vitro* function of the two types of DCs. First, we assessed their ability to phagocytose apoptotic cells, which could affect antigen presentation. Selected or Adherence immature iDCs were cultured with PKH26 stained apoptotic lymphocytes. Cells positive for both HLA-DR staining and PKH26 dye indicated DC phagocytosis of apoptotic cells. 66.4% of Selected and 67% of Adherence iDCs phagocytosed apoptotic cells, indicating no difference in their phagocytic activity (Figure 2B). This process was calcium dependent and inhibited in the presence of EDTA, indicating that apoptotic cells were phagocytosed and not positive for both markers simply by adhering to one another or other non-specific mechanisms.

We next assayed the functional ability of both DC types in an allo-MLR assay. Allogeneic lymphocytes were cultured with Adherence or Selected DCs prepared from PBMC of the same donor. The proliferative response of allogeneic lymphocytes to Adherence DC was not different than their response to Selected DC (Figure 2C, p = 0.483). We then tested the ability of the DC groups to stimulate lymphocyte proliferation in an antigen specific manner. Selected or Adherence DCs were co-cultured with either apoptotic 3T3 cells infected with influenza (DC/flu) or uninfected apoptotic 3T3 cells (DC/ctrl). DC/flu or DC/ctrl were then cultured with syngeneic lymphocytes. Both the Selected and Adherence DCs strongly stimulated influenza-specific lymphocyte proliferation (Figure 2D, p = 0.082 at peak proliferation), again pointing to a similar profile of DC function. Lastly, we assessed the differences in DC ability to induce an IFNγ response in T cells in an antigen specific manner. DC (DC) or influenza infected DC (DCF) were co-cultured with either CD4 or CD8 T cells in an ELISPOT assay. Again, both groups of DCs were able to induce a comparable CD4 and CD8 T cell IFNγ response to influenza (Figure 2E, p = 0.250 and p = 0.822 respectively). In summary, these assays indicate that there is no detectable difference in DC number, phenotype or function that could account for the observation that Adherence DCs produced increased immunogenicity in patients with cancer*.*

### Lymphocytes in Adherence DC vaccine preparations are activated and proliferating

**Figure 3:**
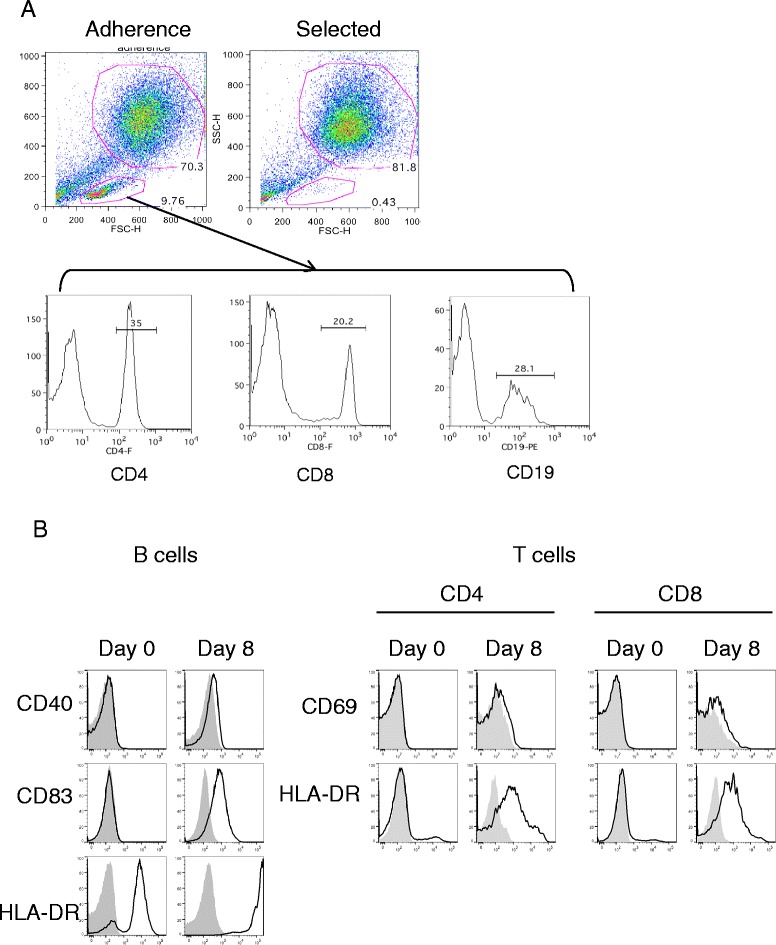
**Lymphocytes in Adherence DC vaccine preparations are activated and proliferating. (A)** Representative flow cytometry profile of forward scatter versus side scatter of Adherence and Selected DC vaccine preparations. CD4, CD8, and CD19 antibody staining of cells in the lymphocytes gate in the Adherence DC plot is shown by histogram. **(B)** DCs made with the Adherence method were stained with antibodies to lymphocyte markers, CD4, CD8 or CD19 and activation markers CD40, CD83, CD69 and HLA-DR and analyzed by flow cytometry. The results shown are representative of 3 different donors.

We next looked beyond the DCs for an explanation for why the two vaccine preparations differed in their ability to stimulate T cell proliferation. Upon examination of the 10 most recent Adherence and Selected DC vaccines administered, we found that Adherence DC vaccines contained a median of 10.8% lymphocytes (range 4.7 to 25.0%, Figure 3A and data not shown), determined by forward and side scatter on flow cytometry. In contrast, the Selected DC vaccines contained very few lymphocytes (median 1.0%, range 0.3 to 1.8%). Thus, we turned our attention to assessment of this population of administered cells. The lymphocytes in the Adherence DC preparation contained a mixture of CD4, CD8 and CD19 cells (Figure 3A). These lymphocyte populations were resting at the time of initial harvest from patients’ blood (Day 0), but were highly activated after 8 days of culture within the DC vaccine preparation. Both CD4 and CD8 T cells up-regulated CD69 and HLA-DR expression and CD19 B cells up-regulated CD40, HLA-DR, and CD83 expression compared to baseline PBMC expression at Day 0 (Figure 3B).

**Figure 4:**
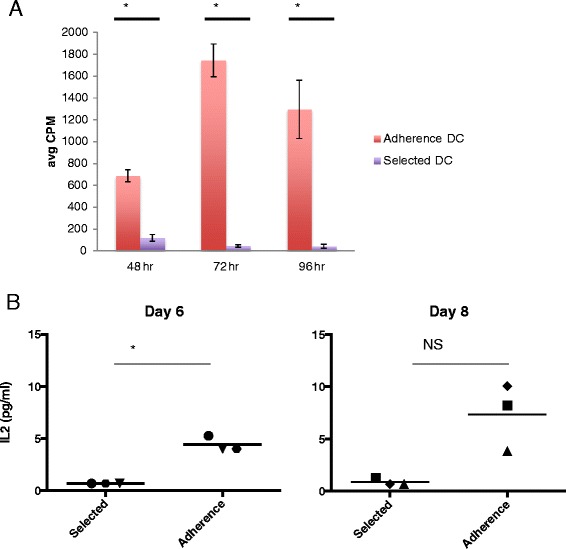
**DC vaccines prepared by Adherence support lymphocyte proliferation and cytokine production. (A)** DCs made by the 2 methods were assessed for proliferation by ^3^H thymidine incorporation at 48, 72, and 96 hours beyond day 8, (the day DCs would have been administered as vaccine). Proliferation responses for Adherence DCs are shown in red and Selected DCs are shown in purple. The results are averages of triplicate wells. * indicates p < 0.05. **(B)** Supernatants of Adherence and Selected DC cultures were examined for pro-inflammatory cytokines on Day 6 and Day 8. Data for IL-2 is shown for 3 donors. Black line represents the mean. * indicates p < 0.05. NS = not statistically significant.

To assess lymphocyte proliferation within the DC preparations, both Adherence and Selected DCs were pulsed with ^3^H-thymidine. Adherence DC preparations were found to have incorporated more ^3^H-thymidine than Selected DCs, an indication that the lymphocytes were proliferating in these cultures (Figure 4A, p = 0.03, p = 0.003, and p = 0.021 respectively at 48, 72, and 96 hours). In addition, when we examined the Adherence and Selected DC cultures for the presence of cytokines after 6 days in culture, we found that the Adherence DC supernatants contained a mean of 4.42 pg/ml IL-2 while the levels in the Selected DC culture supernatants were below the level of detection (<0.68 pg/ml), a statistically significantly increase (Figure 4B, p = 0.012). This supports the observation that lymphocytes present in the Adherence DC culture were proliferating. There was a similar trend after 2 more days of culture in fresh media but this difference was not statistically significant (p = 0.070). There were no differences in the concentrations of IL-8, IL-12p70, IL-1β, IFNγ, IL-6, and IL-10 found in the supernatants of Adherence or Selected DC cultures. In conclusion, Adherence DC vaccines contain both DCs as well as CD4+, CD8+ and CD19+ lymphocytes that are activated *in vitro*, are proliferating, and are producing IL-2.

### DC vaccines prepared by adherence support antigen specific lymphocyte proliferation and activation

Since patient vaccines were prepared by culture of immature (Day 6) DCs with apoptotic tumor cells, we hypothesized that the activated lymphocytes in Adherence DC cultures may expand in an antigen specific manner to apoptotic tumor cells. In this scenario, Adherence DC vaccines could contain an expanded population of tumor cell-specific, activated lymphocytes in addition to DC loaded with tumor cell antigen. Since we did not have enough cancer patient derived specimens to test this directly, we tested the more general hypothesis that lymphocytes contained in Adherence DC preparations proliferate in an antigen specific manner to antigens added to DC cultures on Day 6.

**Figure 5:**
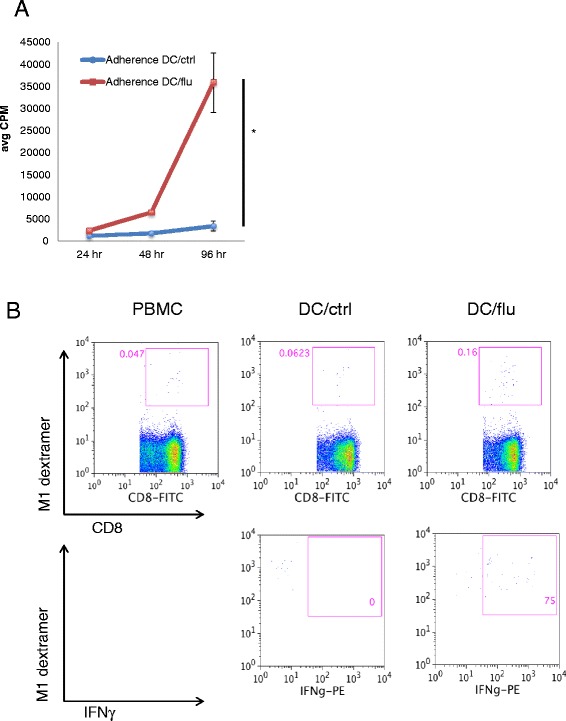
**Proliferation and cytokine production in DC vaccine prepared by adherence method is antigen specific. (A)** Adherence DCs were co-cultured with apoptotic 3T3 (DC/ctrl) or with 3T3 infected with influenza (DC/flu) and assessed for proliferation by ^3^H thymidine incorporation at 24, 48 and 96 hours beyond day 8 (the day DCs would have been administered as a vaccine). Results are of triplicate wells. * indicates p < 0.05. **(B)** Adherence DC/ctrl and DC/flu were stained with CD8 antibody, influenza-M1 specific dextramer and IFNγ antibody. The original PBMC population was used as the comparison group. The upper panels shows CD8+ cells that are gated on M1+ staining. The lower panels show M1+ cells that are gated on IFNγ + cells.

Adherence DCs from healthy donors were co-cultured with either apoptotic influenza infected cell lines (DC/flu) or uninfected control cells (DC/ctrl) and tested for proliferation. There was increased proliferation (Figure 5A, p = 0.009 at 96 hours) in Adherence DC/flu cultures compared to Adherence DC/ctrl cultures. To test whether there were antigen specific CD8+ IFNγ secreting T cells, analogous to those which would be critical for the generation of tumor immunity, we evaluated the cultures for expansion of influenza M1 specific dextramer positive cells. On day 10 of Adherence DC/flu culture, we found an increase in the percentage of influenza M1+ CD8+ cells (0.16%) compared to DC/ctrl cultures (0.0623%) and baseline PBMCs (0.047%, Figure 5B, upper panels). In addition to an increase in the frequency of antigen specific cells, we also found evidence of their activation; on day 10, 75% of the M1-specific CD8 T cells within the DC/flu culture produced IFNγ (Figure 5B, lower panels). Similar results were obtained when this experiment was repeated with a second donor (52% of M1-specific CD8+ T cells produced IFNγ on day 10). Earlier, when we assayed the supernatant for cytokines after 6 and 8 days of culture, no IFNγ was detected due to the lack of antigen. However, when DCs are co-cultured with antigen such as influenza as it was in this culture, not only are we able to detect antigen specific proliferation but we are now able to detect IFN**γ** within that subset. In conclusion, Adherence DC cultures contain a population of proliferating, activated lymphocytes not present in Selected DC cultures and these can expand in an antigen specific manner.

## Discussion

The discovery reported here that a common current method of preparing DCs for clinical vaccine, based on a CD14 selection strategy designed to produce purer populations of DCs, produced antigen-specific T cells with reduced proliferative capacity, was entirely unexpected. A priori, it was hypothesized that purer populations of DCs would be a more robust vaccine strategy. Exploring the mechanism for this difference revealed that DCs prepared by the two methods were comparable in their phenotype and function. We found that the primary difference between the two DC vaccine preparations were the presence of “bystander” lymphocytes in the “less pure” Adherence vaccine preparations; interestingly, we found that not only did these lymphocytes survive in the DC culture but also took on an activated and proliferating phenotype.

One hypothesis is that these activated and proliferating lymphocytes were interacting directly with the DCs to confer Adherence DCs greater potency *in vivo* compared to Selected DCs. For example, it has been shown that survival of DCs can be increased through the TRANCE/TRANCE receptor interaction between a T cell and DC by up-regulation of Bcl-XL expression in the DC [13]. It is possible that the presence of activated T cells in the Adherence DC cultures promoted their survival *in vivo* leading to prolonged antigen-presentation and the increased post-vaccination proliferation we observed in our studies (Figure 1). Further, Berk, et al. showed that iDCs matured with expanding and activated lymphocytes induced greater IL-12p70 and CXCL10 compared to those matured with the conventional cytokine cocktail including TNF-α, IL1, IL6 and PGE2 [14]. Since Adherence DCs are grown with a small number of expanding and activated lymphocytes, these lymphocytes could have a similar enhancing effect on the DCs that potentially contributed to the boosted immunogenicity post-vaccination.

Adherence DC vaccines also contain many CD19+ CD83+ B cells. CD83 is an early activation marker for B cells induced by interaction with activated T cells through CD40 ligation [15,16]. Activated CD83+ B cells are capable of effective tumor-antigen presentation via MHC II [15] and it is possible that B cells in the Adherence DC vaccine potentiate immunogenicity. Moreover, some subset of the immune cells activated by Adherence DCs *in vitro*, including T cells and B cells, might reasonably be expected to be tumor antigen specific, a potentially clinically important observation.

The site of DC administration may be acting as a nidus for *in vivo* lymphocyte recruitment. It has been described that the great majority of DCs administered do not migrate to the draining lymph node but remain at the site of injection [17]. Further, it has been shown that DC/CD4 T cell interactions create local MIP-1α and MIP-1β gradients that attract naïve CD8 T cells to lymph nodes [18]. In the work presented here, we find that lymphocytes in Adherence DC cultures expand and become activated. It is possible that tumor vaccines that contain both DCs and *ex-vivo* activated lymphocytes could incite local inflammation, recruiting additional lymphocytes *in vivo* through such a chemokine gradient even without trafficking to the lymph node.

There are a number of limitations to this work. First, we did not explore what components of the lymphocytes present in the vaccine are necessary for the increased proliferation seen in patients *ex vivo*. Further investigation with additional patients with prostate cancer given different iterations of the vaccine preparation, such as one that contains DCs and CD3+ cells but no CD19+ cells and another that contain DCs with CD19+ cells but no CD3+ cells, for example, are necessary to address this issue. Secondly, we only show head to head comparison made between antigen-specific Adherence and Selected DCs pulsed with apoptotic PC3 cells. It is possible that this difference in immunogenicity is only present under these conditions. We did not explore in detail whether similar differences would be present under other conditions such as with use of alternative sources of antigen, maturation cocktails, route of administration, or in other cancer types. Such investigations are needed to know whether this is a generalizable finding.

DC cancer vaccine trials have resulted in varied immunological and clinical responses [19]. It is becoming increasingly clear that DCs may require adjuvants and this is demonstrated by the recent breakthroughs using CTLA4 and PD1 blockade [20,21]. While CD14 magnetic selection generates a highly pure population of precursor monocytes, the data presented here demonstrate that purity may actually hinder potency. Since there was no detectable difference in the cell surface phenotype or *in vitro* function of Adherence and Selected DC besides the presence of activated lymphocytes, we propose that these lymphocytes provide a needed adjuvant effect. Of note, the only DC tumor vaccine approved by the FDA, Sipuleucel-T, is generated by isolating cells with density gradient centrifugation and is therefore highly unlikely to contain DCs exclusively. It is possible that the activity of this agent is in part due to the presence of activated lymphocytes [22]. Future studies will focus on dissecting which signals from activated lymphocytes are necessary and sufficient to optimize DC vaccines.

## Conclusions

A surprising difference found in the immunogenicity of DC cancer vaccines made by two different methods of DC preparation prompted us to look into the underlying causes. We found that the dose of DCs administered, DC phenotype, and viability was similar in both vaccine types given. In vitro DC function as assessed by allo-MLR and proliferation assays were also similar. However, we show that DCs generated by the Adherence method are accompanied by proliferating and activated lymphocytes. We hypothesize that the presence of activated lymphocytes may contribute to the enhanced responses seen in patients given vaccine made using this preparation method. This is an important finding, given the variety of methods used in preparing DC vaccine studies and the mixed post-vaccination responses seen. As we evaluate DC vaccines in the future, it is imperative that we consider methods of DC preparation and how this may play a role in post-vaccination responses. Further investigation is warranted with more patients and with other variables, as this finding could have important ramifications for future DC vaccine preparation strategies.

## Additional file

Additional file 1: Figure S1. Description of data: The pre- and post-vaccination proliferation responses to PC3 and LNCaP are shown for all patients. Raw proliferation response data are shown.
