# Supplementary figures and images for: Dendritic cell vaccines containing lymphocytes produce improved immunogenicity in patients with cancer
Source: J Transl Med. 2014 Dec 5;12:338. doi: 10.1186/s12967-014-0338-3 (PMC4264264; doi:10.1186/s12967-014-0338-3)

## Slide 1
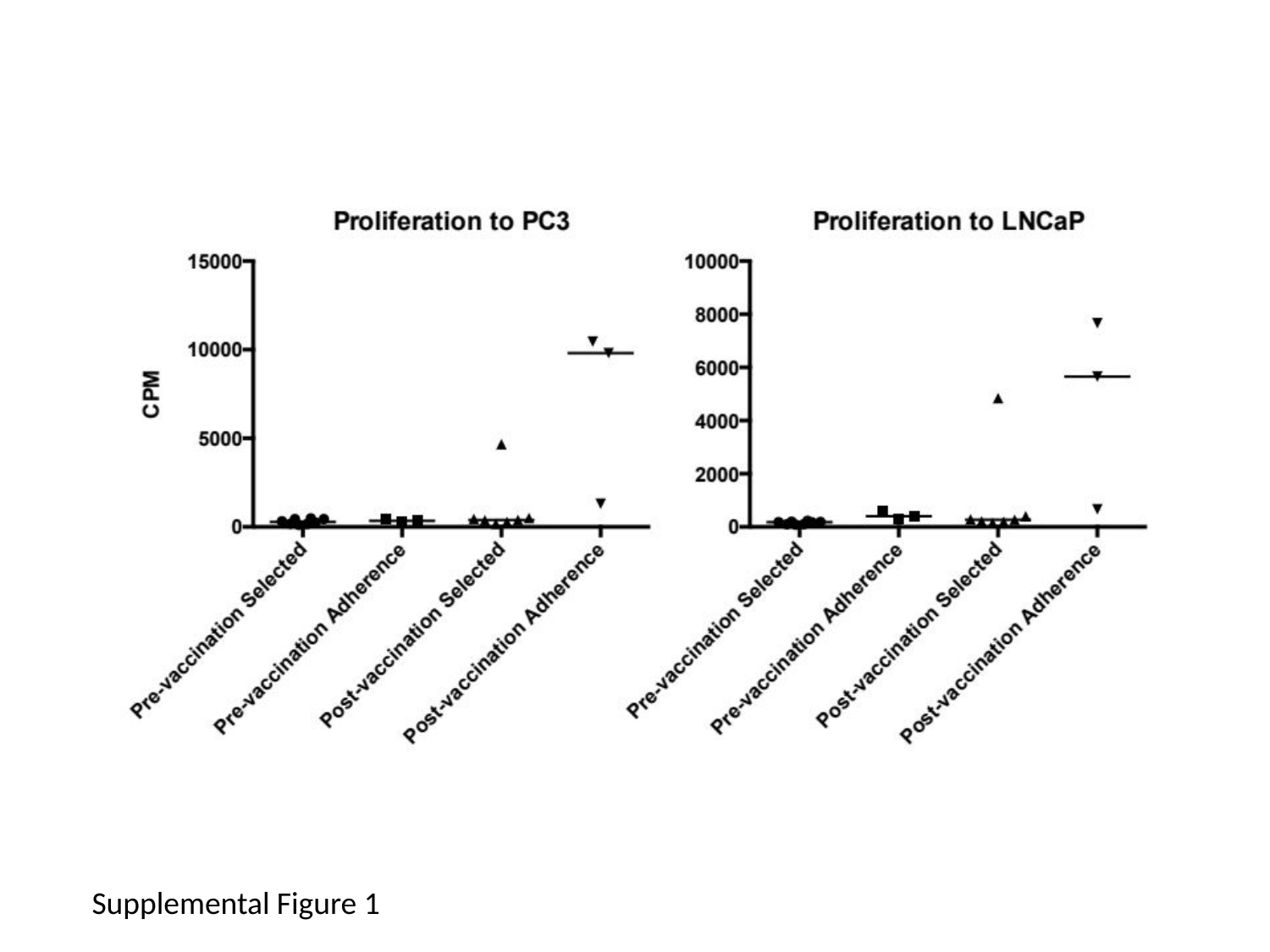

Supplemental Figure 1

Supplement: Additional file 1: Figure S1. — Description of data: The pre- and post-vaccination proliferation responses to PC3 and LNCaP are shown for all patients. Raw proliferation response data are shown. [file 12967_2014_338_MOESM1_ESM.pptx]
